# Quaternary Ice Ages Shaped Protists Phylogeography: The Case of Arcellinida in the Iberian Peninsula

**DOI:** 10.1111/mec.70475

**Published:** 2026-07-18

**Authors:** Rubén González‐Miguéns, Emilio Cano, Enrique Lara

**Affiliations:** ^1^ Real Jardín Botánico (RJB‐CSIC) Madrid Spain; ^2^ Institut de Biologia Evolutiva (CSIC‐Universitat Pompeu Fabra) Barcelona Spain; ^3^ Research Support Unit Real Jardín Botánico (CSIC) Madrid Spain

**Keywords:** Amoebozoa, biogeography, evolution, microbiology

## Abstract

The Quaternary glaciations profoundly shaped the biogeography of plants and animals, yet their impact on microbial eukaryotes remains largely unexplored. We tested the “genetic legacy of the Quaternary” (GLQ) paradigm in terrestrial protists using Arcellinida testate amoebae diversity distribution across the Iberian Peninsula, a well‐established glacial refugium. To do so, we compiled the most extensive Arcellinida metabarcoding dataset to date (ArKOI), including 615 samples from multiple continents and ecosystems. Our results showed that hotspots of intra‐OTU genetic diversity align with known Iberian refugia, supporting the concept of “refugia within refugia,” and displaying clear ecoregion‐specific climatic niches. Patterns of spatial clustering, niche breadth, and historical demographic reconstruction revealed repeated range contractions and expansions during the Pleistocene, mirroring those observed in macro‐organisms. These findings extend the GLQ paradigm to protists, highlighting shared historical and ecological processes across the eukaryotic tree of life and contributing to a unified theory of biogeographic responses to climatic change.

## 1 Introduction

Species geographic distributions and diversification patterns are strongly influenced by historical climatic and geological processes that shape the spatial structure of biodiversity (Sanmartín 2012). Among the best‐characterized of these events are the Quaternary glacial cycles (between 2.6 Ma and 11.7 ka before present), which have profoundly influenced the distribution and genetic spatial structuring of animals and plants. This succession of warm and cold periods left a trace widely referred to as the “genetic legacy of the Quaternary ice ages” (GLQ hereafter) (Taberlet et al. 1998; G. Hewitt 2000; Schmitt 2007; Dapporto et al. 2024). While the effects of the GLQ on the contemporary biogeography of many macroscopic taxa have been widely studied, comparable knowledge is lacking for microbial‐sized organisms. This gap is particularly evident when considering protists, eukaryotic microorganisms that are neither animals, nor fungi, nor plants but make up the largest part of eukaryotic diversity (Burki et al. 2021). Consequently, our limited understanding of protistan biogeographic patterns in an evolutionary framework hinders both the extension of the GLQ paradigm to Domain Eukarya as a whole, which would allow a generalized framework to test biogeographic hypotheses grounded on global historical events.

During the Quaternary, repeated glacial–interglacial cycles drove major shifts in the distribution of polar ice sheets, mountain glaciers, and associated biomes, profoundly reshaping biodiversity patterns across Europe and the Iberian Peninsula. At a local scale, regional topography or the proximity of warm water masses left glacial refugia; zones with milder temperatures, reduced ice cover, or higher water availability, that allowed local populations to survive (G. Hewitt 2000). Outside these refugia, populations were extirpated, or at least drastically reduced, undergoing genetic bottlenecks. During milder climatic episodes, glacial refugia behaved as sources for the recolonization of surrounding territories, acting as biogeographic “islands.” In Europe, the three Mediterranean Peninsulas (i.e., Iberian, Italic and Balkanic) acted as glacial refugia, and were the starting point for northward expansion routes. Eventually, these range expansions produced secondary contact zones between closely related taxa (Taberlet et al. 1998; Schmitt 2007), leaving a lasting imprint on the present‐day inter‐and intraspecific genetic diversity of many organisms. Among the three major refuge zones, the Iberian Peninsula stands out for its pronounced topographic and ecological climatic heterogeneity. As such, it harbours multiple, internally isolated refugia (appropriately called “refugia within refugia”), generating complex patterns of biodiversity distributions and secondary‐contact zones (Gómez and Lunt 2007; Médail and Diadema 2009; Abellán and Svenning 2014). The extensive comparative data available for plants and animals, coupled with the high density of distinct internal refugia within a relatively small area (~580,000 km^2^), make the Iberian Peninsula an outstanding natural setting for testing the GLQ paradigm in protists.

Terrestrial species are often preferred for testing historical biogeographic hypotheses because freshwater systems are generally more spatially constrained by drainage‐independent barriers (Rahel 2007). Within protists, however, diversity data on terrestrial assemblages are still scarce, leaving only a handful of taxa with sufficient data to evaluate specific historical hypotheses (Geisen et al. 2018). One of the best‐documented terrestrial protist groups is the order Arcellinida (lobose testate amoebae). This group has played a central role in debates surrounding microbial cosmopolitanism and the “everything is everywhere” paradigm (Whitfield 2005), which suggests that the immense dispersal capacity of protists allows their fast global spread, regardless of geographical barriers. A more recent phylogeographic study has suggested a probable impact of glacial cycles on the distribution of a species complex within Arcellinida in the Holarctic realm (Singer et al. 2019), raising the possibility that historical climatic oscillations also shaped contemporary diversity patterns in terrestrial microbial eukaryotes. Furthermore, their systematics and taxonomy are relatively well resolved (González‐Miguéns, Todorov, et al. 2022; Porfirio‐Sousa et al. 2024), and group‐specific metabarcoding protocols are available (Ruggiero et al. 2020; González‐Miguéns et al. 2023). Together, these characteristics make Arcellinida an excellent model system for testing ecological and biogeographic hypotheses, including the GLQ paradigm, in continental environments.

Accordingly, our goal was to test whether terrestrial protists exhibit biogeographic patterns consistent with the GLQ paradigm, which predicts that Pleistocene climatic oscillations promoted population isolation in refugial areas followed by postglacial expansion dynamics. To address this question, we generated *de novo* soil metabarcoding data from across the Peninsula and integrated them with all Arcellinida metabarcoding datasets published to date into a standardized database, ArKOI. This comprehensive resource allowed us to evaluate three main predictions of the GLQ paradigm in these terrestrial protists. First, under the GLQ framework, intraspecific genetic diversity is expected to show a geographically structured distribution, with diversity hotspots concentrated in regions previously identified as glacial refugia for macroorganisms. Second, populations are expected to show demographic signatures associated with Quaternary climatic oscillations, including signals compatible with population bottlenecks, isolation, and subsequent postglacial expansions. Third, if climatic stability during glacial cycles influenced the persistence of Arcellinida populations, species distributions and genetic diversity should also display associations with distinct climatic niches across Mediterranean and Temperate ecoregions. We hypothesize that, despite their microscopic size and potentially high dispersal capacity, Arcellinida biogeographic patterns follow processes comparable to those observed in macroorganisms. If supported, these results would extend the GLQ framework to microbial eukaryotes and reinforce the possibility that common historical mechanisms structure biodiversity across the whole Domain Eukarya.

## 2 Materials and Methods

### 2.1 eDNA Extraction, Amplification and Sequencing

118 Soil samples were collected across the Iberian Peninsula (Spain and Portugal) and Europe (France, Ireland, Czech Republic, and Switzerland) (Supporting Information S1). Environmental DNA (eDNA) extraction, amplification, and sequencing were performed as described in previous studies (González‐Miguéns et al. 2023) and followed the recommendations (Goldberg et al. 2016).

We amplified a portion of 407 bp from the cytochrome oxidase subunit I (COI), using a two‐step semi‐nested PCR protocol specifically designed for Arcellinida. For the first PCR, we used the universal COI primers pair LCO‐1490 as forward and HCO‐2198 as reverse (Folmer et al. 1994), and for the second PCR, we used LCO‐1490 as the forward primer and the Arcellinida‐specific primer ArCOIR as reverse (González‐Miguéns et al. 2021). In order to sort sequence reads by sample (demultiplexing), we used a unique combination of primers with tags for each sample in this second PCR, as described in (González‐Miguéns et al. 2023). Finally, we proceeded to sequencing using a MiSeq 500 cycles v2 paired‐end 250 bp. All sequencing was performed in the Genomics Unit of the Fundación Parque Científico de Madrid, Spain (PRJNA1476739).

### 2.2 ArKoi Database

As an initial step toward testing the “genetic legacy of the Quaternary ice ages” (GLQ hereafter (G. Hewitt 2000; Dapporto et al. 2024)) in the Iberian Peninsula (Gómez and Lunt 2007), we assembled a comprehensive database of Arcellinida metabarcoding studies. Six published metabarcoding datasets were retrieved (Supporting Information S1), We then added *de novo* metabarcoding data from the Iberian Peninsula terrestrial samples described above, and re‐assembled with a standardized pipeline. We selected metabarcoding studies specifically targeting Arcellinida that utilized the ArCOI primers (Supporting Information S1). For each of these studies, raw sequence data were independently assembled de novo following the standardized protocol and format described in (González‐Miguéns et al. 2026). The reads per sample were processed using the DADA2 package in R (Callahan et al. 2016). After obtaining the ASVs and read counts per locality, tag‐jumping artefacts were removed using the custom script script_0 (Supporting Information).

All localities were assigned to an ecoregion based on “WWF Terrestrial Ecoregions Of The World (Biomes)” and abiotic variables derived from WorldClim (Fick and Hijmans 2017, 2) (accessed on 19/03/2025), using shapefiles (.shp) from these sources and the latitude/longitude of each sample.

All individual FASTA files retrieved from the different metabarcoding studies were merged into a single file named ArKOI_database.fasta following (González‐Miguéns et al. 2026).

Finally, ASV taxonomic assignment was conducted using the eKOI taxonomy database (González‐Miguéns et al. 2025), implemented through a custom script (5_taxonomic_assignation.py). ASVs showing less than 84% similarity to reference Arcellinida sequences were excluded as non‐Arcellinida. This threshold was determined from local phylogenetic reconstructions and taxonomically validated single‐cell reference datasets (González‐Miguéns et al. 2023, 2024; Useros et al. 2025), corresponding to the maximum divergence at which sequences could still be confidently assigned to Arcellinida. Although conservative, and therefore potentially excluding some undescribed Arcellinida diversity, this approach minimizes the inclusion of non‐Arcellinida sequences arising from the limited taxonomic coverage currently available for environmental protist datasets. Subsequently, a FASTA file containing only Arcellinida ASVs was generated, forming the final ArKOI database (Supporting Information S2). A final FASTA file was generated, integrating ASVs across localities and aligned using MAFFT (v7.490) (Katoh and Standley 2013). Finally, VSEARCH was employed to cluster ASVs at a 97% identity threshold, a value previously shown to be a good approximation to delineate Arcellinida species (González‐Miguéns et al. 2024; García‐Bodelón et al. 2025), to form OTUs.

### 2.3 Intra‐OTU Diversity Per Sample

We evaluated the spatial distribution of the ratio between OTUs and ASVs taxonomically assigned to Arcellinida obtained from terrestrial samples from the Iberian Peninsula. Following (González‐Miguéns et al. 2024), the OTU/ASV ratio was used as a proxy for within‐OTU genetic diversity, where ASVs represent fine‐scale sequence variants (=haplotypes) nested within broader OTU clusters (an approximation for species). For each locality, the ratio was calculated as the number of ASVs divided by the number of OTUs detected in the sample, thus estimating the average level of infra‐OTU genetic variation in the sample. Lower OTU/ASV ratios therefore indicate higher haplotype diversity within OTUs (equivalent to high intraspecific diversity). Under the GLQ framework, regions acting as long‐term refugia are expected to retain higher intra‐OTU genetic diversity due to prolonged lineage persistence, whereas recently recolonized regions are expected to exhibit reduced haplotypic variation (Petit et al. 2003). Localities with an OTU/ASV ratio < 0.5 were analysed with a kernel‐density estimate (KDE) using the script “script_1” (Supporting Information). To assess spatial structure, we constructed a k‐nearest‐neighbours matrix (*k* = 4) and computed global spatial autocorrelation with Moran's I, using 1000 Monte‐Carlo permutations. Moran's I was also calculated separately for each ecoregion.

Then to ensure robust biogeographical analyses and minimize potential sequencing artefacts, we filtered the data to retain only informative OTUs, defined as those (1) containing at least four sequences from a minimum of two distinct locations and (2) containing at least one pair of sequences that differ by ≥ 1% (uncorrected p‐distance), using the K80 model, following (González‐Miguéns et al. 2026) (Supporting Information S4). The objective of this filtering strategy was not taxonomic selection per se, but to retain OTUs with sufficient spatial and haplotypic resolution for meaningful phylogeographic inference.

### 2.4 Population Structure and Ecoregion Characterization Per Informative OTU

Next, we investigated the intra‐OTU diversity structuring for each informative OTU. We built haplotype networks using the haploNet function (Paradis 2010), based on Minimum Spanning Networks (MSNs). Then, we examined genetic structuring both among and within ecoregions using a modified haplotypic‐diversity index, an inversed PH_d_ (Population Haplotype Diversity) (González‐Miguéns et al. 2026). PH_d_ extends the traditional haplotype diversity metric (H_d_) by incorporating the relative spatial distribution of haplotypes among sites, here defined as ecoregions. The relative frequency of each haplotype within an OTU was calculated as its diversity within a given ecoregion divided by its total diversity across the entire distribution of that OTU. PH_d_ therefore quantifies the extent to which genetic diversity is spatially concentrated or partitioned among ecoregions, allowing inference of population‐level genetic structuring from metabarcoding haplotype data. Lower PH_d_ values indicate stronger geographic structuring of haplotypes, whereas higher values indicate more homogeneous haplotype distributions among ecoregions, with one dominant ecoregion. For each informative OTU, the ecoregion contributing the largest relative share of haplotypes was considered as the putative source population. We selected the informative OTUs to be kept for further analyses based on their PH_d_ on a series of filters, as follows: (i) Ecosystem filter; we retained only those informative OTUs with the highest Ph_d_ in terrestrial ecosystems; and (ii) Geographic filter; from this subset, we selected only OTUs with the highest Ph_d_ in the Iberian Peninsula. Finally, we performed an ecoregion assignment (Temperate or Mediterranean) of the terrestrial Iberian informative OTUs according to their PH_d_ values. Additionally, we calculated nucleotide diversity (π) using the *nuc.div* function (Paradis 2010). To evaluate whether informative OTUs showed significant genetic structuring among ecoregions inferred from PH_d_, we performed analyses of molecular variance (AMOVA) using script_2 (Supporting Information). For each of these OTUs individually, we (i) calculated a pairwise genetic‐distance matrix among haplotypes and (ii) performed a hierarchical AMOVA with ecoregion and sampling site as nested spatial factors (ecoregion/site), using 999 permutations to assess statistical significance. This analysis allowed us to quantify the proportion of genetic variation explained by differences among ecoregions relative to within‐site variation.

### 2.5 Spatial Distribution of Informative OTUs Across the Iberian Peninsula

To visualize the spatial distribution of haplotype richness and the degree of geographic sharing among ecoregions for informative OTUs, we calculated, for each sampling locality, the proportion of haplotypes that were unique to each ecoregion or shared among multiple ecoregions (using script_3_a; Supporting Information). These proportions were represented as pie charts to illustrate the relative contribution and overlap of haplotypes across regions. Additionally, kernel‐density contours were generated separately for each ecoregion to visualize the spatial concentration of haplotype diversity.

We then mapped the haplotype networks per informative OTUs in the Iberian Peninsula map, using the script script_3_c (Supporting Information). Nucleotide diversity (π) was calculated per locality for each OTU (with respect to the haplotypes shared in the locality by each informative OTU) and used to colour the haplotypes in the geographic map. A background KDE (Kernel‐Density Estimate) was performed with the function kde2d, based on localities with π ≥ 0.005. The haplotype networks of all informative OTUs were covered: haplotype links are coloured by genetic distance, and dotted when a haplotype occurs in multiple localities (Figure 3C,D). The resulting figure simultaneously shows sampling effort, genetic connectivity among haplotypes, and within site molecular diversity.

We tested whether haplotype richness per informative OTU was associated with geographic range size within each ecoregion. For each OTU, all occurrence coordinates within a given ecoregion were used to construct a minimum convex polygon (convex hull), and its area (km^2^) was calculated as a proxy for geographic range size using the script script_S5 (Supporting Information). Haplotype richness and estimated range size were then compared using Spearman's rank correlation (*cor.test* in R), separately for Mediterranean and Temperate ecoregions.

### 2.6 Niche Characterization of the Informative OTUs

The Iberian Peninsula is characterized by steep climatic gradients, and diversity dynamic patterns are conditioned by each OTU's ecological tolerance. We characterized the environmental niche of each informative OTU of the Iberian Peninsula by extracting the 19 WorldClim bioclimatic variables together with elevation for all occurrence localities (Fick and Hijmans 2017, 2), as proxies for climatic niche. In that purpose, we used the script ‘script_4_a_b’ (Supporting Information).

To characterize the overall environmental differentiation between ecoregions, we first calculated climatic centroids (mean values of all environmental variables) for each informative OTU and performed a principal components analysis (PCA) including all OTUs from both Mediterranean and Temperate ecoregions together. This global PCA provided a common environmental background allowing direct comparison between ecoregions. Environmental variables differing significantly between ecoregions were further evaluated using one‐way ANOVAs. To explore environmental structuring within each ecoregion, separate PCAs were then performed independently for Mediterranean and Temperate datasets. Because these PCAs were calibrated independently, their axes are not directly comparable between ecoregions, but instead describe the main environmental gradients within each regional climatic space.

We then projected haplotype networks into ‘climatic space’ to integrate ecological and genetic information using script_4_c_d (Supporting Information). For each ecoregion separately, PCA spaces were constructed using the abiotic variables associated with sampling localities containing informative OTU haplotypes. Individual localities were represented as points in climatic space, with colours representing the nucleotide diversity (π), while connections among points reproduced the haplotype‐network relationships among localities belonging to the same OTU. Line colour was scaled according to pairwise genetic distance between connected haplotypes. This approach allowed visualization of whether genetically similar haplotypes occupied similar portions of environmental space within each ecoregion.

### 2.7 Evaluating Correlation Between Niche Breadth and Genetic Distances in Both Ecoregions

We estimated ecological niche breadth for every informative OTU in each ecoregion with the script script_5_a (Supporting Information). For all ASVs belonging to a given OTU, climatic variables were extracted from occurrence localities. Niche breadth was then quantified as the mean standard deviation across all environmental variables, such that higher values indicate occupation of a broader range of climatic conditions, whereas lower values reflect narrower environmental tolerances. Mean genetic distances per ecoregion were calculated with the script script_5_b (Supporting Information), which computes the average pairwise genetic distance for each informative OTU within each ecoregion. OTUs with mean intra‐OTU divergence below 1% were interpreted as compatible with the Refuge model (R model), characterized by recent demographic expansion and limited genetic structuring, whereas OTUs exceeding this threshold were considered compatible with the Sanctuary model (S model), consistent with long‐term persistence and deeper diversification within refugial areas (Recuero and García‐París 2011).

### 2.8 Niche Overlap Among OTUs and Between Ecoregions

We quantified niche overlap between informative OTUs with the script script_5_c (Supporting Information). Abiotic variables were first reduced to a three‐axis PCA space (PC1–PC3), using PCA. For every informative OTU, we generated a Gaussian hypervolume that describes its climatic niche (Blonder et al. 2014). Pairwise niche overlap among OTUs was quantified using Sørensen, Jaccard, and unique‐fraction indices derived from hypervolume overlap. To further evaluate whether observed niche overlap differed from random expectations, we used the random points generated within each hypervolume as environmental background (González‐Miguéns, Soler‐Zamora, et al. 2022). Using the ecospat R package (Di Cola et al. 2017), we projected pairwise OTU combinations onto 100 × 100 environmental grids and performed both niche‐equivalency and niche‐similarity tests with 100 permutations (Warren et al. 2008). These analyses allowed us to assess whether OTUs occupied statistically equivalent climatic niches or whether observed similarities could arise under random environmental use.

To evaluate overlap at the ecoregion level we used script_5_d (Supporting Information). Each sampling site was assigned to one of three categories: (1) sites containing a single informative OTU (‘alone’); (2) sites containing multiple informative OTUs assigned to the same ecoregion; and (3) sites containing multiple informative OTUs associated with different ecoregions. For each informative OTU, we then calculated the proportion of occurrences falling within each category to assess patterns of spatial coexistence and ecoregion overlap.

### 2.9 COI Molecular Clock and Extended Bayesian Skyline Plot

To place our analyses in a temporal framework, we derived a molecular clock rate from a well‐documented diversification event, the inception of the *Hyalosphenia papilio* species complex. This clade is a group of Arcellinida strictly confined to *Sphagnum* bogs that are closely associated with these ecosystems, approximately between 7 and 20 Ma ago (Shaw et al. 2010; Bechteler et al. 2023), in the Miocene. Because diversification within the *H. papilio* complex is thought to be linked to the emergence of these habitats, this system provides one of the few available temporal calibration points for Arcellinida evolution.

COI sequences from several of *H. papilio* lineages (Singer et al. 2019) were downloaded and analysed in BEAST v1.10.5 (Drummond and Bouckaert 2015) to calibrate a molecular clock for Arcellinida. Four independent runs were conducted, each with a different clock model: (1) classical random‐local, (2) shrinkage‐local, (3) strict, and (4) uncorrelated relaxed. All runs employed a GTR substitution model with estimated base frequencies, and the root age was constrained to 7 Ma using a log‐normal prior (mean = 7, SD = 0.1), following (Singer et al. 2019). Posterior distributions of *clock. rate*, *branch rates, shrinkage. rate*, and *mean rate* substitution‐rate parameters were sampled after removing the first 20% of generations as burn‐in, and mean substitution‐rate estimates across models were averaged to obtain a single COI clock estimate for Arcellinida. The resulting rate was compared with published COI clocks for other metazoan phyla (Supporting Information S5) and subsequently used to calibrate Extended Bayesian Skyline Plot analyses in BEAST 2 v2.7.7 (Bouckaert et al. 2019), applying the strict‐clock model separately to each informative OTU.

## 3 Results

### 3.1 ArKOI Database

**FIGURE 1:**
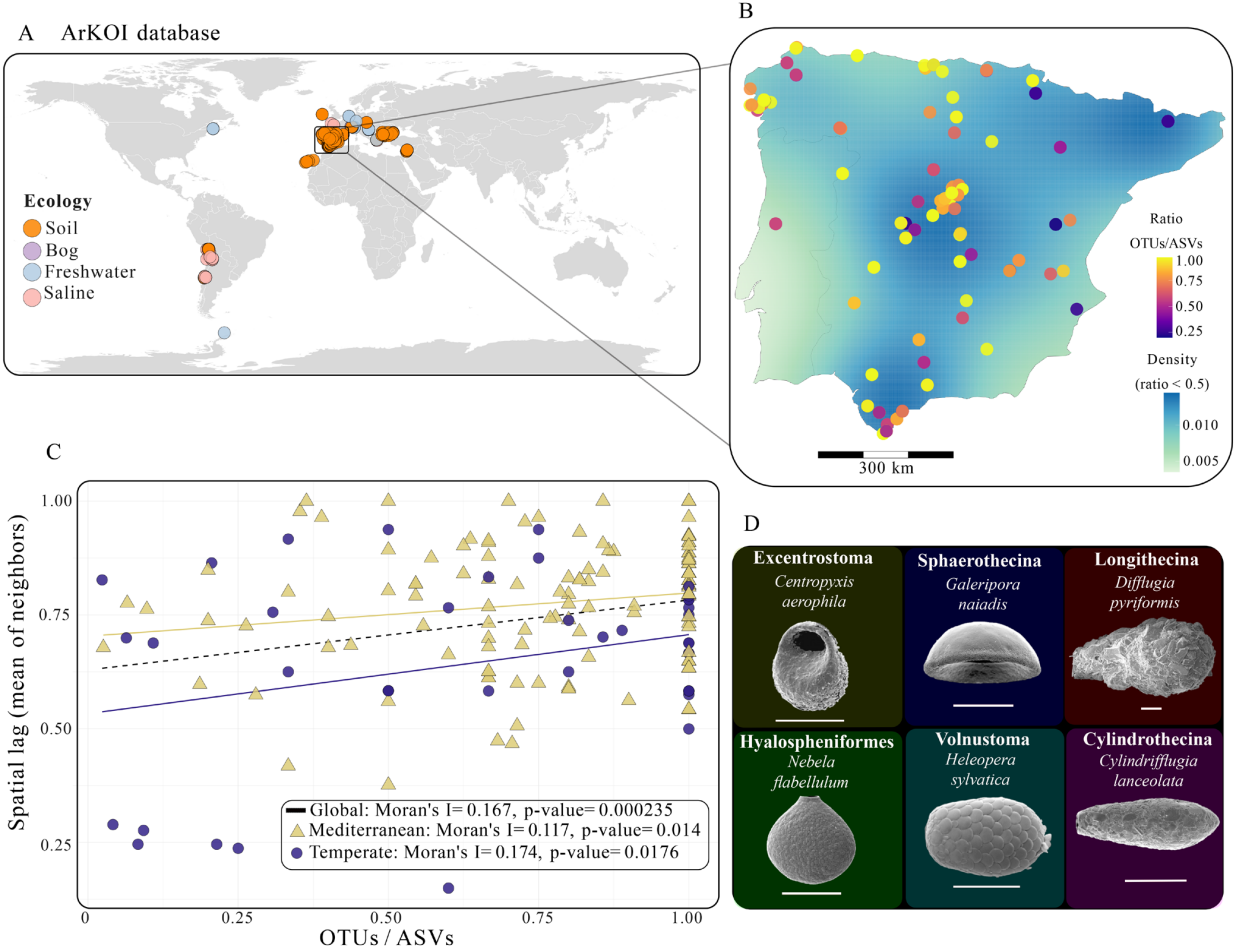
Global coverage of ArKOI and spatial autocorrelation of OTU/ASV ratios in terrestrial Iberian soils. (A) ArKOI database: Global map showing every sample currently included in the ArKOI database, colour‐coded by ecosystem type. (B) Iberian Peninsula soil points (KDE samples with ratio < 0.5): Terrestrial samples from the Iberian Peninsula coloured by their OTU/ASV ratio; a kernel‐density estimate (KDE) surface highlights diversity hotspots based on samples with ratios ≥ 0.05. (C) Spatial lag versus ratio by ecoregions in the Iberian Peninsula: Moran's *I* values for OTU/ASV ratios calculated for all Iberian terrestrial samples combined, and each ecoregion analysed separately. (D) Representative scanning‐electron micrographs of the major Arcellinida morphotypes; images modified from (González‐Miguéns, Todorov, et al. 2022). Scale bars = 50 μm.

The resulting database, called ArKOI (Supporting Information S2), contains 615 samples, with 17,023 Arcellinida amplicon sequence variants (ASVs) supported by 26,717,017 reads, clustered into 5398 operational taxonomic units (OTUs) (Supporting Information S3) (Figure 1A). ArKOI database spans freshwater, soil and saline continental ecosystems across Europe, the Americas and Western Asia. This standardized database provides a robust framework for testing biogeographical, ecological, and evolutionary hypotheses in Arcellinida. Within the Iberian Peninsula alone, 274 samples produced 2830 Arcellinida ASVs (1,394,795 reads) clustered into 1217 OTUs (Figure 1B). Each sample was assigned to an ecoregion (Olson et al. 2001), That is, the Iberian Peninsula, (1) Mediterranean Forests, Woodlands (Mediterranean, for short); and (2) Temperate Broadleaf and Mixed Forests (Temperate).

If intra‐OTU molecular diversity is shaped by long‐term climatic stability during Quaternary glacial cycles, it should display non‐random geographic structuring across the Iberian Peninsula, with diversity hotspots concentrated in regions that acted as climatic refugia. To evaluate this, we tested for spatial autocorrelation in the OTUs/ASVs ratio of Arcellinida (González‐Miguéns et al. 2024). Across all Iberian samples we detected a significant, positive spatial autocorrelation with the ratio (Moran's I = 0.1668, *p* = 0.00023; Monte‐Carlo observed = 0.16684, *p* = 0.001), indicating that sites with similar ratio values tend to be geographically close to each other (Figure 1B,C). Both the Mediterranean (Moran's *I* = 0.117, *p* = 0.014) and the Temperate ecoregion (Moran's *I* = 0.174, *p* = 0.0176) showed significant positive autocorrelation (Figure 1C). The consistent clustering of samples with elevated intra‐OTU molecular diversity is consistent with the existence of geographically structured hotspots of intra‐OTU diversity hotspots in the Peninsula, a result that is compatible with the existence of Pleistocene climatic refugia as postulated in the GLQ paradigm in macroorganims (G. Hewitt 2000; Dapporto et al. 2024). Although the observed effect sizes are moderate, the consistent positive spatial autocorrelation across both ecoregions suggests that intra‐OTU diversity is not randomly distributed across the Iberian Peninsula.

### 3.2 Informative OTUs From Terrestrial Ecosystems in the Iberian Peninsula

In order to delimit accurately the location of the biodiversity hotspots, we restricted the analyses to a selection of ‘informative OTUs’. Across the complete ArKOI dataset, we recovered 341 informative OTUs (Supporting Information S4). We then identified which of these OTUs exhibited the highest levels of haplotypic diversity in terrestrial ecosystems of the Iberian Peninsula. In based to the PH_d_ metric (González‐Miguéns et al. 2026), we identified 32 OTUs whose highest haplotype diversity occurred in Iberian terrestrial systems. All belonged to the infraorder Excentrostoma, the most diverse Arcellinida clade in terrestrial environments (González‐Miguéns et al. 2024; ElKhouri‐Vidarte et al. 2025). Among these OTUs, 26 showed their highest PH_d_ values in the Mediterranean ecoregion, whereas 6 peaked in the Temperate ecoregion. Most informative OTUs displayed median PH_d_ values close to zero, indicating limited spatial partitioning of haplotypes among ecoregions. In general, haplotypes tended to be predominantly associated with a single ecoregion rather than evenly distributed across both climatic regions, a pattern that was further supported by AMOVA analyses (Figure S1).

### 3.3 Geographical Spatial Structuring of Informative Species Across the Iberian Peninsula

**FIGURE 2:**
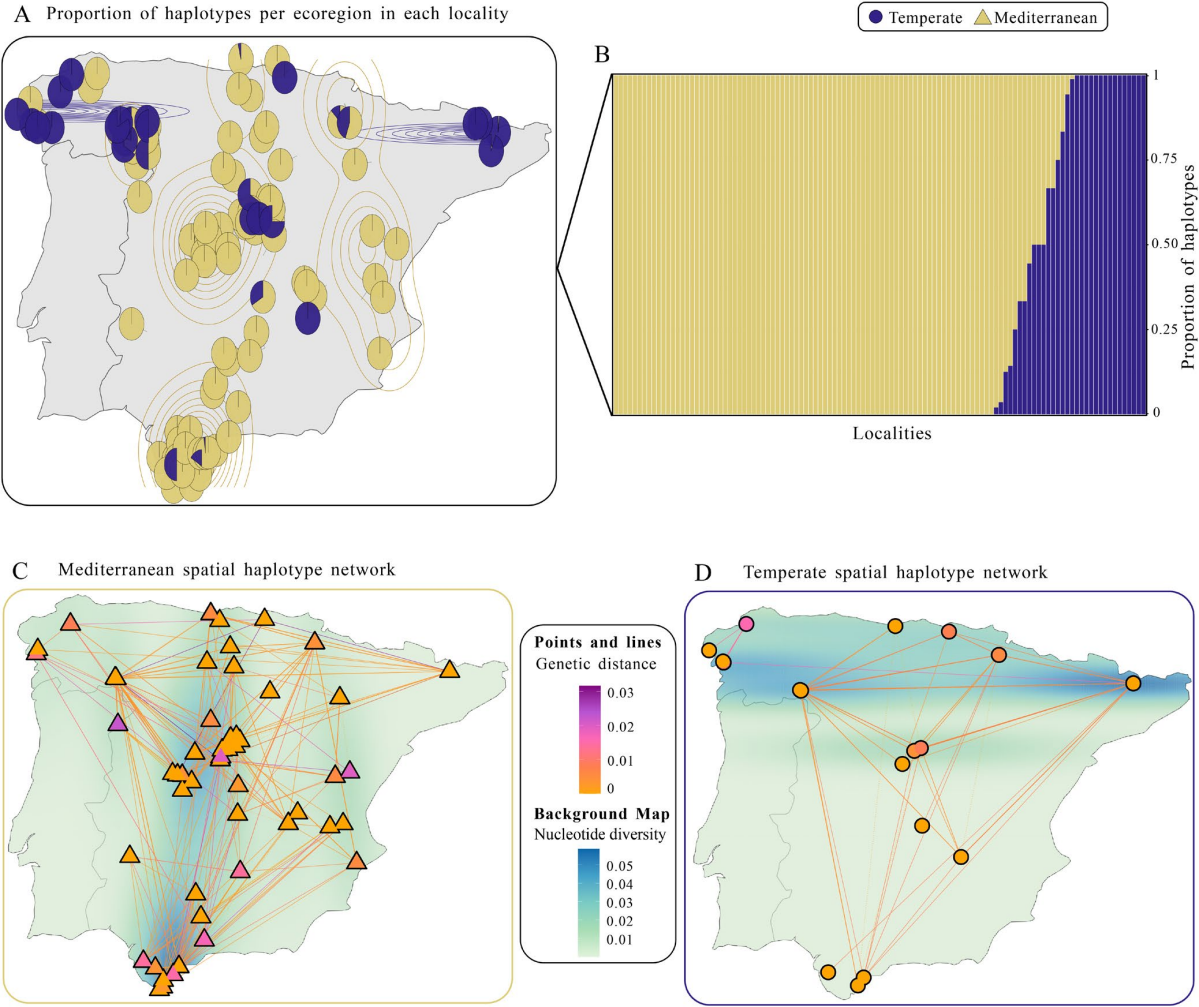
Intra‐OTU molecular structuring across the Iberian Peninsula. (A) Relative frequency of haplotypes per ecoregion in samples containing informative OTUs; smooth curves depict kernel‐density estimates of the total number of haplotypes per ecoregion. (B) Boxplots showing the proportion of haplotypes per ecoregion for each locality that harbours informative OTUs. (C) For every Mediterranean informative OTU, maps plot haplotype composition by Iberian locality, with point colour indicating nucleotide diversity among haplotypes of each OTU in that locality; connecting lines follow OTU‐specific haplotype networks, where colour reflects pairwise genetic distance and dashed lines mark identical haplotypes shared by multiple localities; background colour of the map represents the kde of localities with pi > = 0.005 (D) Same representation as in (C) for informative OTUs from the Temperate ecoregion.

We examined how the genetic diversity of these 32 informative OTUs is distributed geographically across both ecoregions in the Iberian Peninsula. First, we mapped haplotype richness and the proportion of haplotype overlap for every Iberian sample. Mediterranean informative OTUs showed their highest haplotype richness in three areas: the ‘Sistema Central’, the Baetic region, and the Iberian System (Figures 2a, S2 and S3). The Temperate informative OTUs display two hotspots of haplotypic diversity in the north‐western (Galaic‐Portuguese) and north‐eastern (Catalonian Pyrenees) corners of the peninsula (Figure 2A, S1 and S2). Most localities sampled in the Iberian Peninsula contained haplotypes belonging only to OTUs from the same ecoregion; the co‐occurrence between Mediterranean and Temperate OTUs was rare and occurred only in 17 out of the 274 surveyed sites (Figure 2B).

We used a complementary approach to corroborate the observed intra‐OTU spatial patterns. In that purpose, we combined haplotype‐network topology with per‐sample nucleotide diversity. The resulting patterns mirrored those based on haplotype richness, with the single exception of the diversity hotspot found in the Iberian System, which was no longer detected. Still, we observed two clear clusters of molecular diversity emerging among the Mediterranean OTUs located in the Central System and Baetic region, respectively (Figures 2C and S2). The haplotypes found in these clusters were connected to surrounding localities by relatively low genetic distances, consistent with geographically structured diversification and local dispersal. Temperate OTUs retained the same two northern clusters (Galaic‐Portuguese and Catalonian Pyrenees), with peripheral haplotypes likewise linked by low molecular divergence (Figures 2D and S3). Together, these spatial patterns of intra‐OTU diversity broadly coincided with the locations of well‐characterized Pleistocene climatic refugia previously identified for animals and plants on the Iberian Peninsula (Gómez and Lunt 2007; Abellán and Svenning 2014).

### 3.4 Climatic‐Niche Structuring of Terrestrial Informative OTUs Across the Iberian Peninsula

**FIGURE 3:**
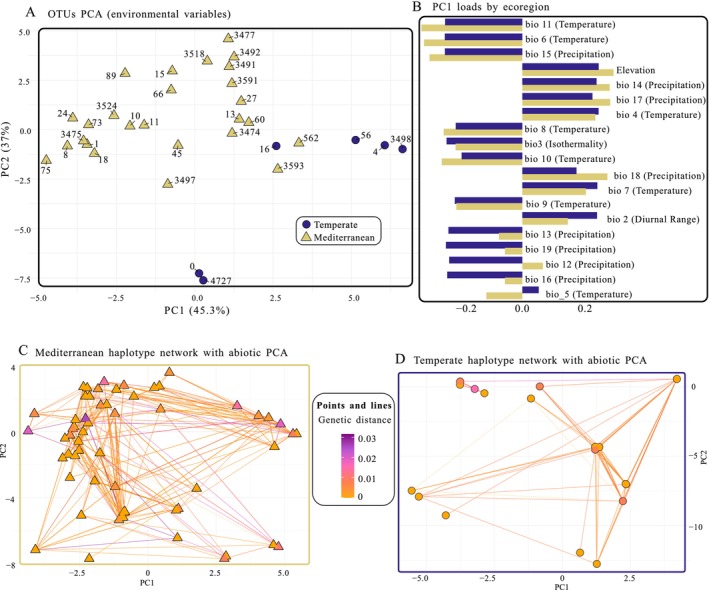
Climatic niche characterization of Iberian informative OTUs by ecoregion. (A) Principal‐components analysis (PCA) of all informative OTUs based on 19 bioclimatic variables plus elevation; points are coloured by ecoregion and the five longest loading vectors highlight the most influential variables. (B) Variable loadings on PC1, shown separately for Mediterranean and Temperate informative OTUs. (C) Mediterranean climatic‐space PCA showing sampling localities containing informative OTU haplotypes. Points represent localities coloured according to nucleotide diversity (π) of the corresponding OTU at each site. Connecting lines represent haplotype‐network relationships among localities belonging to the same OTU, with line colour proportional to pairwise genetic distance. Dashed lines indicate identical haplotypes shared among multiple localities. (D) Same visualization as in (C) for informative OTUs from the Temperate ecoregion.

Having identified intra‐OTU molecular diversity hotspots for the 32 Iberian informative OTUs, we aimed at identifying which current climatic factors are associated with their present‐day spatial distribution. In particular, we investigated which climatic factors might have acted differently on the Mediterranean and Temperate informative OTUs, respectively, in order to better characterize their phylogeographic history. We ran a PCA on the 19 bioclimatic variables plus elevation provided by WorldClim (Fick and Hijmans 2017, 2) for every informative OTU. The ordination separated clearly Mediterranean and Temperate OTUs (Figure 3A). Precipitation‐related variables contributed most strongly to PC1, which explains 45.3% of the variance (Figure 3B). Furthermore, a one‐way Analysis of variance (ANOVA) performed on each abiotic variable revealed that OTU niches were mainly driven by factors related to precipitation in both ecoregions, and temperature for Mediterranean ecoregion (Figure S4). Such a result is in line with previous works that emphasize humidity as one of the most influential variables in shaping Arcellinida niches (Singer et al. 2019; Useros et al. 2023; ElKhouri‐Vidarte et al. 2025).

We obtained a more detailed picture of the distribution of intra‐OTU diversity by combining haplotype‐network topology with per‐sample nucleotide diversity onto the same climatic PCA. Mediterranean OTUs occupy a broader portion of climatic space (Figure 3C). Most haplotypes fell into one or two dense clusters that harboured most of the haplotypic diversity within each OTU; peripheral haplotypes were connected with low genetic distances from these centres and contained little additional nucleotide diversity. In contrast, Temperate OTUs had a noticeably narrower climatic space than Mediterranean OTUs (Figure 3D). Like for the Mediterranean ecoregion, diversity for each OTU is concentrated into one or two clusters; however, the spread of peripheral haplotypes is noticeably smaller, consistent with the comparatively narrower climatic space characterizing the Temperate ecoregion. These results reinforce the view that climate plays a key role in shaping the spatial structuring of Arcellinida lineages, suggesting distinct climatic optima between ecoregions.

### 3.5 Climatic Niche Overlap Between Mediterranean and Temperate Informative Species

**FIGURE 4:**
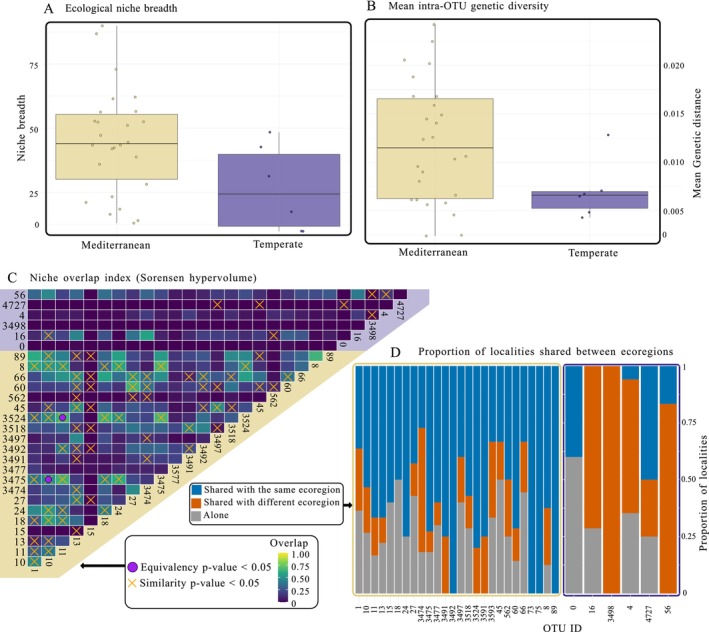
Climatic‐niche and geographic overlap among Iberian informative OTUs by ecoregion. (A) plot of niche breadth for each Iberian informative OTU, grouped by ecoregion. (B) plot of the mean intra‐OTU geographic distance for each Iberian informative OTU, grouped by ecoregion. (C) Pairwise Sørensen indices of climatic‐niche overlap based on hypervolumes calculated for each informative OTU; crosses indicate niche similarity significantly higher than expected by chance, whereas filled circles denote full niche equivalence. (D) Boxplot showing the percentage of Iberian localities in which each informative OTU co‐occurs with haplotypes of another informative OTU from a different ecoregion, co‐occurs with an OTU from the same ecoregion, or occurs alone.

Given the contrasting patterns of genetic diversity observed between Mediterranean and Temperate OTUs, we hypothesized that both groups may reflect different demographic histories and post‐glacial range dynamics, which could be reflected in the degree of climatic niche overlap among informative OTUs. As a first step, we compared the niche breadth and mean intra‐OTU genetic diversity of the 32 Iberian informative OTUs using Iberian records only. Mediterranean OTUs showed markedly larger median niche hypervolumes (43.94) and longer mean intra‐OTU genetic distances (0.01149) than their Temperate counterparts (24.30 and 0.0066, respectively; Figure 4A,B). The Spearman rank test revealed a positive correlation between geographical range and haplotype number in Mediterranean OTUs (*ρ* = 0.47, *p* = 0.016; Figure S5). In contrast, such a relationship was not significant in Temperate OTUs (*ρ* = −0.14, *p* = 0.80; Figure S5), consistent with a stronger effect of demographic bottlenecks or range restriction in Temperate lineages.

To calculate the degree of climatic niche overlap between OTUs, we generated hypervolumes from PCA axes of the climatic variables described above, revealing substantially greater niche overlap among OTUs belonging to the same ecoregion than between ecoregions (Figure 4C). Both niche‐equivalence and niche‐similarity tests confirmed that climatic niches were significantly more similar than expected by chance, primarily among OTUs belonging to the same ecoregion. Finally, the ‘locality‐sharing’ analysis (Figure 4D) showed that most Temperate OTUs co‐occurred with Mediterranean lineages, whereas Mediterranean OTUs co‐occurred predominantly with other Mediterranean OTUs. This asymmetric co‐occurrence pattern is consistent with the hypothesis that Temperate OTUs occupy a comparatively narrower climatic and geographic subset within the broader Mediterranean environmental space, potentially reflecting different post‐glacial demographic and range‐expansion dynamics.

### 3.6 Temporal Context in the Iberian Terrestrial Informative OTUs

**FIGURE 5:**
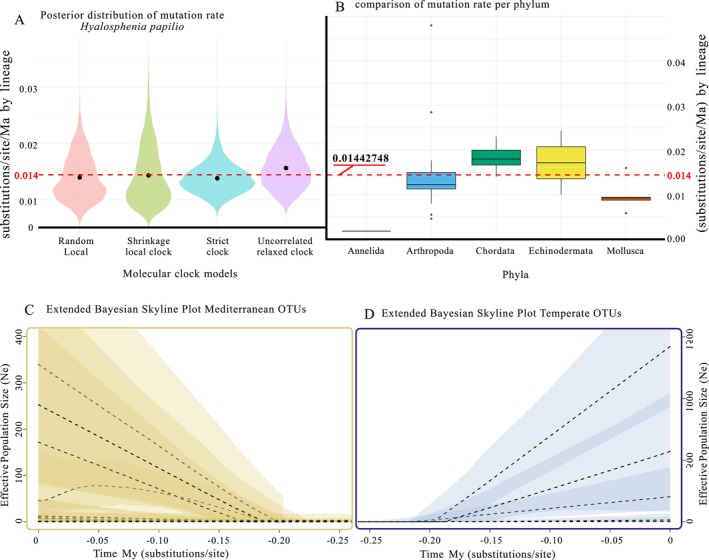
Temporal framework for Arcellinida. (A) Violin plots of branch‐specific substitution rates (subs/site^−1^/Myr^−1^) estimated under each molecular‐clock model; the dashed horizontal line marks the mean across clocks. (B) Comparative box‐plot of branch substitution rates for major metazoan phyla, plotted alongside the mean Arcellinida rate (0.014). (C) Extended Bayesian Skyline Plot (EBSP) showing demographic history of Mediterranean informative OTUs: Dashed lines, median effective population size; shaded envelopes, 95% highest‐posterior density. (D) Same EBSP representation for informative OTUs from the Temperate ecoregion.

A temporal framework is still necessary in order to determine the implication of Pleistocene climate shifts in present‐day Arcellinida spatial molecular diversity patterns. Because no standard COI substitution rate exists for Arcellinida, we used as a reference date the emergence of the *H. papilio* species complex, corresponding with the development of the boreal *Sphagnum* peatlands to which it is confined (Singer et al. 2019). Assuming the diversification of these amoebae followed the inception of boreal raised bogs (between 7 and 20 Ma ago (Shaw et al. 2010; Bechteler et al. 2023)), we dated COI trees under several molecular‐clock models. The median of the models converged on ~0.014 substitutions site^−1^ Myr^−1^ within lineage (Figure 5A), a value comparable to estimates for most animal phyla (Papadopoulou et al. 2010) (Supporting Information S5; Figure 5B). Although no truly universal clock exists, this rate provides a useful proxy for placing diversification and demographic events on an approximate timescale in Arcellinida COI.

Using this rate in Extended Bayesian Skyline Plot (EBSP) analyses under a strict‐clock model, we reconstructed relative demographic trajectories through time for each OTU (Figure 5C,D). Importantly, the inferred effective population sizes (Ne) should not be interpreted as absolute demographic estimates for Arcellinida populations. Still, recent population genomic studies suggest that Arcellinida may have lower‐than‐expected effective population sizes that might be congruent with our values despite large census abundances (Weiner et al. 2023). Rather, EBSP analyses are used here primarily to infer relative temporal changes in lineage dynamics under a coalescent framework. Given the use of a single short mitochondrial marker (~360 bp), together with the difficulty of translating coalescent estimates into biologically realistic Ne values for microbial eukaryotes, absolute Ne estimates remain highly uncertain. Despite these limitations, where temporal resolution permitted, major demographic shifts clustered around ~200 ka, within the Pleistocene. Although similar single‐marker phylogeographic approaches were originally developed mainly for metazoan systems, the temporal patterns recovered here are broadly consistent with demographic responses expected under Quaternary climatic oscillations.

Interestingly, Temperate OTUs displayed higher inferred coalescent Ne values than Mediterranean OTUs, despite exhibiting lower haplotypic diversity and narrower climatic niches. This apparent discrepancy suggests that nucleotide diversity and coalescent‐based demographic estimates may capture different aspects of population history and therefore may not scale linearly. In particular, most Temperate OTUs are characterized by low intra‐OTU genetic diversity and fit predominantly within the Sanctuary (S) model, consistent with stronger historical bottlenecks and restricted diversification (see discussion). In contrast, Mediterranean OTUs generally exhibit higher haplotypic diversity and broader climatic distributions, being more frequently associated with the Refuge (R) model. These contrasting demographic histories may help explain why Mediterranean lineages retain higher contemporary genetic diversity despite lower inferred coalescent Ne values. Overall, rather than focusing on the absolute magnitude of Ne estimates, we interpret these analyses as evidence that both Mediterranean and Temperate Arcellinida lineages persisted through Pleistocene climatic oscillations while experiencing distinct demographic dynamics.

## 4 Discussion

### 4.1 Geographical Structuring of Potential Iberian Refuges for Arcellinida

Our results indicate that the genetic structuring of the different Arcellinida OTUs aligns with Pleistocene climatic refugia previously characterized for multicellular plants and animals. This demonstrates that the GLQ paradigm can also apply to at least some unicellular microorganisms. Furthermore, our findings support the ‘refugia within refugia’ hypothesis in Arcellinida that states that the Iberian Peninsula, which constitutes a refuge as a whole, includes distinct, independent centres of high intraspecific molecular diversity (Olalde et al. 2002; Gómez and Lunt 2007). We identified potential climatic refugia for Temperate and Mediterranean ecoregions, respectively.

For the Temperate ecoregion, we identified two main areas of high intra‐OTU diversity (Figures 2D and S3): (i) the Galaic‐Portuguese region in the west, which has acted as a refuge for taxa that expanded their distribution during the Holocene, such as, for instance, the Iberian frog *Rana iberica* (Teixeira et al. 2018) or the pedunculated oak *Quercus robur* (Olalde et al. 2002); (ii) the Eastern Pyrenean region in the east, similarly for organisms as the viviparous lizard *Zootoca vivipara* (Horreo et al. 2018) and the downy oak *Quercus pubescens* (Olalde et al. 2002).

For the Mediterranean ecoregion, we identified two areas of high intra‐OTU diversity that likely acted as Pleistocene climatic refugia for Arcellinida (Figures 2C and S2): (i) the “Sistema Central” (Central system), corresponding to the Guadarrama and Gredos mountain ranges in the centre of the Peninsula, acting as refugia for organisms such as the Iberian green lizard *Lacerta schreiberi* (Godinho et al. 2008) or the midwife toads of genus *Alytes* (Lucati et al. 2022)*;* (ii) the Baetic Range, in southern Iberia, harbouring species such as *Timon lepidus* (Miraldo et al. 2011), and the microscopic rotifer *Brachionus manjavacas* (Gómez et al. 2007) among others.

### 4.2 Sanctuaries vs. Climatic Refuges in the Iberian Peninsula for Arcellinida

Pleistocene phylogeographic patterns depend on each taxon life history and are therefore known to be species‐specific, still they can be broadly classified into two models (Recuero and García‐París 2011): (i) the Sanctuary model (S model), where Pleistocene glaciations likely caused population fragmentation and promoted lineage sorting, but allowed the preservation of part of the ancestral diversity accumulated before the glaciations; and (ii) the Refuge model (R model), where only a small portion of the ancestral population survived in the climatic refugia, and most of the ancestral intraspecific diversity was lost. In the S‐model, larger genetic distances can be expected. In turn, in the R‐model, a much shallower genetical diversification is expected due to demographic bottlenecks associated with population contraction during glacial periods, followed by post‐glacial expansion from a reduced number of surviving lineages. Furthermore, if different populations have remained isolated in separate refugia, the geographic contact zones where these lineages meet during post‐glacial expansion may accumulate elevated mitochondrial diversity due to the coexistence of divergent lineages originating from different refugial areas (Petit et al. 2003). However, with mitochondrial markers alone, distinguishing between long‐term persistence within refugia and secondary contact among expanding lineages remains difficult.

When applying a threshold of > 1% mean intra‐OTU divergence to classify OTUs according to refugial models (Figure 5B), we found that, among the Temperate ecoregion OTUs, only one out of six meets the criteria for the Sanctuary model (S model). The temperate zone of the Iberian Peninsula is primarily a colonization area for species from temperate Europe, representing the southern range limits of many taxa. These species may have had their centre of diversity in higher latitudes, but these populations may have been extirpated. This peripheral distribution likely explains the dominance of R model patterns in this ecoregion in the Iberian Peninsula. In contrast, 15 out of 26 Mediterranean OTUs would fall within the S model (Figure 5B). These OTUs also exhibit a more heterogeneous pattern of genetic structuring, consistent with long‐term persistence in multiple refugial areas across the Iberian Peninsula, with distinct centres of higher molecular diversity corresponding to the climatic refugia. These patterns suggest that the genetic diversity of populations may be more strongly influenced by their position relative to historical refugia than by their present‐day distribution range.

### 4.3 Arcellinida Climate Niche Facing the Future Challenge of Climate Change

Our results confirm that wide scale climate changes play a major role in shaping the geographical structuring of intraspecific diversity in Arcellinida. Precipitations is the primary factor shaping the current geographic distribution of OTUs from the temperate ecoregion (Figures 4A,B and S3). Previous studies have demonstrated that humidity is one of the most important variables in the biogeography of protists in general (Bates et al. 2013; Geisen et al. 2018). In Arcellinida, depth to water table (a proxy for humidity) is one of the most relevant diversity drivers in peatlands; moreover, transitions between aquatic and terrestrial ecosystems are rare, having occurred only a few times in Arcellinida evolutionary history (Useros et al. 2023, 2025). In contrast, temperature‐related variables were also important drivers for those OTUs associated with the Mediterranean ecoregion. This suggests that the distribution of Arcellinida OTUs is shaped not only by fine‐scale microhabitats but also by broader climatic conditions, implying that the ongoing global warming could profoundly impact the distribution and population dynamics of these species.

One of the greatest challenges of the Anthropocene is climate change, marked by rising global temperatures (Steffen et al. 2011). The Iberian Peninsula is projected to be one of the most strongly affected regions in Europe, with significant temperature increases and precipitation declines leading to progressive aridification (Carvalho et al. 2021). Under these conditions, there is evidence of a migration of (macroscopic) species into higher latitudes/altitudes within very short evolutionary timescales (Benito Garzón et al. 2008; Chen et al. 2011). Conversely, species adapted to temperate climates are experiencing population declines, in effective size, ecological function, and geographic distribution (Thuiller et al. 2006; Benito Garzón et al. 2008), placing them at risk of extinction (Urban 2024). The distribution patterns observed for Arcellinida OTUs mirror these trends described for animals and plants. Temperate ecoregion OTUs exhibit more restricted distributions and narrower niche breadths, compared to Mediterranean ecoregion OTUs (Figure 5A,B). These Mediterranean OTUs are also found, although in relatively low proportions, in the northern Iberian Peninsula (within the temperate zone). They present low nucleotide diversity in northern localities, and have close genetic similarity to southern populations (see previous discussion), which suggests that their presence is likely the result of recent colonization events. Thus, our results suggest that Mediterranean Arcellinida OTUs are in a process of expansion toward the northern, temperate regions of the Iberian Peninsula, consistent with broader patterns of climate‐driven range shifts.

## 5 Conclusions

Our study has several limitations. Phylogeographic inferences are based on a single mitochondrial marker, which captures only part of the evolutionary history of populations. Likewise, the demographic reconstructions derived from short mitochondrial fragments should be interpreted primarily as relative demographic trajectories rather than as absolute estimates of effective population size (Ne). Finally, although we detected a broad geographic correspondence between hotspots of intra‐OTU diversity and refugial regions described for macroorganisms, the identification of refugial areas and contact zones in protists remains necessarily coarse at the scale of the Iberian Peninsula. Future studies integrating higher‐resolution molecular data and finer‐scale geographic sampling will be required to characterize these regions more precisely and evaluate the molecular consequences of secondary contact among lineages.

Despite these limitations, our results demonstrate that Pleistocene climatic oscillations have left a persistent imprint on the intraspecific genetic structure of Arcellinida, revealing striking parallels with phylogeographic patterns long described in plants and animals (G. Hewitt 2000, 2004; Dapporto et al. 2024). The concordance between Arcellinida intra‐OTU structuring and well‐established Iberian refugial regions supports the extension of the GLQ paradigm to unicellular eukaryotes and provides clear evidence for a ‘refugia within refugia’ scenario in the Iberian Peninsula. Moreover, the differential climatic drivers shaping Arcellinida distributions, precipitation in temperate regions and temperature in Mediterranean ones, underscore the sensitivity of these taxa to large‐scale climatic gradients. Finally, situating our demographic inferences within the well‐established timeline of Quaternary glacial–interglacial cycles (G. Hewitt 2000, 2004), we can confirm the implication of QLC in Arcellinida, and possibly other protists groups. Such results suggest a generalization of QLC to the Domain Eukarya as a whole, no matter the size of the organisms.

## Author Contributions

**Rubén González‐Miguéns:** conceptualization, investigation, methodology, formal analysis, data curation, validation, visualization, supervision, writing – original draft, writing – review and editing. **Emilio Cano:** methodology, writing – review and editing. **Enrique Lara:** conceptualization, methodology, writing – original draft, writing – review and editing, supervision, resources, project administration, funding acquisition.

## Funding

This work was funded by as a grant from the Spanish Government (Program Generación de Conocimiento) PID2021‐128499NB‐I00, https://doi.org/10.13039/501100011033 (MCIU/AEI/FEDER, UE), as well as another grant from the National Plan for Scientific and Technical Research and Innovation (PN2022—Research Consolidation—State Subprogram for Incorporation—State Programme to Develop, Attract and Retain Talent) https://doi.org/10.13039/501100011033.S125 (E.L.). We also acknowledge the grant JDC2023‐050439‐I funded by MICIU/AEI/https://doi.org/10.13039/501100011033 and by ESF+ (R.G.‐M.).

## Conflicts of Interest

The authors declare no conflicts of interest.

## Supporting information


**Supporting Information: S1** Metadata table for the ArKOI database, including sampling localities, ecological information, and the studies from which the raw sequence data were obtained.

**Supporting Information: S2** ArKOI database in FASTA format, with each sequence header containing information on read abundance and the locality where the sequence was detected.

**Supporting Information: S3** Text file specifying the OTU assignment of each sequence included in the ArKOI database.

**Supporting Information: S4** Table summarizing the results of the different models applied in this study for each informative OTU.

**Supporting Information: S5** Substitution rates (site^−1^ Myr^−1^) reported for published COI molecular clocks in some metazoan phyla.

**Figure S1:** Informative terrestrial OTUs in the Iberian Peninsula. (A) Percentage of molecular variance explained by ecoregion (AMOVA): Analysis of molecular variance (AMOVA) for each informative OTU, showing the percentage of molecular variance attributable to ecoregion. (B) PHd of each OTU per ecoregion: Results of the PHd analysis for the informative OTUs, summarized by ecoregion.
**Figure S2:** Geographic distribution of each OTU present in the Mediterranean ecoregion of the Iberian Peninsula. Each panel shows a map with the localities where the corresponding OTU (number shown above) was detected. Points represent sampling localities and are coloured according to nucleotide diversity (π) estimated for that OTU in that locality, following the colour scale shown
**Figure S3:** Geographic distribution of each OTU present in the Temperate ecoregion of the Iberian Peninsula. Each panel shows a map with the localities where the corresponding OTU (number shown above) was detected. Points represent sampling localities and are coloured according to nucleotide diversity (π) estimated for that OTU in that locality, following the colour scale shown
**Figure S4:** Results of one‐way ANOVAs for environmental variables from WorldClim that differed significantly between the Mediterranean and Temperate ecoregions. Variables are ranked by F‐statistic and displayed as a horizontal bar plot. Variable names follow the WorldClim bioclimatic codes (bio).
**Figure S5:** Relationship between haplotype richness and geographic range size for informative OTUs in the Mediterranean and Temperate ecoregions. Geographic range was estimated as the area (km2) of a convex hull enclosing all occurrence records for each OTU, based on coordinate data. Haplotype richness and range size were analysed separately for each ecoregion using Spearman's rank correlation. Points represent OTUs (Mediterranean: yellow triangles; Temperate: blue circles), with dashed lines showing fitted trends. Spearman's *ρ* and *p*‐values are indicated for each ecoregion.

## Data Availability

All data, code, results, including ArKOI metabarcoding database, are available via github (https://github.com/rubenmiguens/Arcellinida_GLQ), Figshare (https://doi.org/10.6084/m9.figshare.29885813.v1), and in the Supporting Information. Raw sequence data are accessible in GenBank with the accession PRJNA1476739.
