# Supplementary material for: Quaternary Ice Ages Shaped Protists Phylogeography: The Case of Arcellinida in the Iberian Peninsula
Source: Mol Ecol. 2026 Jul 18;35(14):e70475. doi: 10.1111/mec.70475 (PMC13380319; doi:10.1111/mec.70475)
Supplement: Supplementary file 5 — Supporting Information: S5 Substitution rates (site−1 Myr−1) reported for published COI molecular clocks in some metazoan phyla. [file MEC-35-e70475-s005.pdf]

Supplementary data S5: Substitution rates (site^{-1} Myr^{-1}) reported for published COI molecular clocks in some metazoan phyla.

Min	Max	Mean	Phylum	Study
0.0067	0.0108	0.00875	Mollusca	[1](Ketmaier et al., 2010)
-	-	0.0125	Arthropoda	[2](Ketmaier et al., 2003)
-	-	0.0177	Arthropoda	[3](Papadopoulou et al., 2010)
-	-	0.0115	Arthropoda	[4](Brower, 1994)
-	-	0.01	Arthropoda	[5](Juan et al., 1995)
0.004	0.007	0.0055	Arthropoda	[6](Andersen et al., 2000)
-	-	0.0285	Arthropoda	[7](Clarke et al., 2001)
-	-	0.015	Arthropoda	[8](Farrell, 2001)
0.0092	0.014	0.0116	Arthropoda	[9](Quek et al., 2004)
0.006	0.01	0.008	Arthropoda	[10](Pfeiler et al., 2006)
0.0115	0.0155	0.0135	Arthropoda	[11](Kiyoshi & Sota, 2006)
-	-	0.016	Arthropoda	[12](Sota & Hayashi, 2007)
0.012	0.013	0.0125	Arthropoda	[13](Caccone & Sbordon, 2001)
-	-	0.012	Arthropoda	[14](Percy et al., 2004)
-	-	0.0167	Arthropoda	[15](Pons & Vogler, 2005)
0.013	0.017	0.015	Arthropoda	[16](Wahlberg, 2006)
-	-	0.0046	Arthropoda	[17](Ruiz et al., 2009)
-	-	0.048	Arthropoda	[18](Gratton et al., 2008)
0.0168	0.0293	0.02305	Chordata	[19](Patané et al., 2009)
-	-	0.00582	Mollusca	[20](Fourdrilis et al., 2016)
0.0113	0.0145	0.0134	Arthropoda	[21](Andújar et al., 2012)
0.007	0.018	0.0112	Arthropoda	[22](Marshall et al., 2016)
0.01	0.022	0.016	Mollusca	[23](García-Ríos et al., 2014)
0.0067	0.0121	0.0094	Mollusca	[24](Marko, 2002)

-	-	0.018	Chordata	[25](Lavinia et al., 2016)
-	-	0.0115	Arthropoda	[26](Saltzwedel et al., 2017)
0.00677	0.0118	0.009285	Arthropoda	[27](Hupalo et al., 2020)
-	-	0.0115	Arthropoda	[28](Wang et al., 2014)
-	-	0.014	Chordata	[29](Khan et al., 2014)
0.009	0.011	0.01	Echinodermata	[30](Foltz et al., 2008)
0.014	0.0347	0.02435	Echinodermata	[31](Loeza-Quintana & Adamowicz, 2018)
-	-	0.02	Chordata	[32](Pellegrino et al., 2014)
0.0075	0.011	0.00925	Mollusca	[33](Ketmaier et al., 2006)
-	-	0.01165	Arthropoda	[34](Schubart et al., 1998)
-	-	0.01665	Chordata	[35](Soares et al., 2009)
0.0013	0.0023	0.0018	Annelida	[36](Chevaldonné et al., 2002)

References

1. Ketmaier, V., Manganelli, G. & Tiedemann, R. Peri-Tyrrhenian Phylogeography in the Land Snail Solatopupa guidoni (Pulmonata). *Malacologia* **52**, 81–96 (2010).
2. Ketmaier, V., Argano, R. & Caccone, A. Phylogeography and molecular rates of subterranean aquatic Stenasellid Isopods with a peri-Tyrrhenian distribution. *Molecular Ecology* **12**, 547–555 (2003).
3. Papadopoulou, A., Anastasiou, I. & Vogler, A. P. Revisiting the Insect Mitochondrial Molecular Clock: The Mid-Aegean Trench Calibration. *Molecular Biology and Evolution* **27**, 1659–1672 (2010).

4. Brower, A. Phylogeny of *Heliconius* Butterflies Inferred from Mitochondrial DNA Sequences (Lepidoptera: Nymphalidae). *Molecular Phylogenetics and Evolution* **3**, 159–174 (1994).
5. Juan, C., Oromí, P. & Hewitt, G. Mitochondrial DNA Phylogeny and Sequential Colonization of Canary Islands by Darkling Beetles of the Genus Pimelia (Tenebrionidae). *Proceedings. Biological sciences / The Royal Society* **261**, 173–80 (1995).
6. Andersen, N. M., Cheng, L., Damgaard, J. & Sperling, F. A. H. Mitochondrial DNA sequence variation and phylogeography of oceanic insects (Hemiptera: Gerridae: Halobates spp.). *Marine Biology* **136**, 421–430 (2000).
7. Clarke, T. E., Levin, D. B., Kavanaugh, D. H. & Reimchen, T. E. RAPID EVOLUTION IN THE NEBRIA GREGARIA GROUP (COLEOPTERA: CARABIDAE) AND THE PALEOGEOGRAPHY OF THE QUEEN CHARLOTTE ISLANDS. *Evolution* **55**, 1408–1418 (2001).
8. Farrell, B. D. Evolutionary Assembly of the Milkweed Fauna: Cytochrome Oxidase I and the Age of TetraopesBeetles. *Molecular Phylogenetics and Evolution* **18**, 467–478 (2001).
9. Quek, S.-P., Davies, S. J., Itino, T. & Pierce, N. E. Codiversification in an Ant-Plant Mutualism: Stem Texture and the Evolution of Host Use in Crematogaster (Formicidae: Myrmicinae) Inhabitants of Macaranga (Euphorbiaceae). *Evolution* **58**, 554–570 (2004).
10. Pfeiler, E., Bitler, B. G., Ramsey, J. M., Palacios-Cardiel, C. & Markow, T. A. Genetic variation, population structure, and phylogenetic relationships of *Triatoma rubida* and *T. recurva* (Hemiptera: Reduviidae: Triatominae) from the Sonoran Desert, insect vectors of the Chagas' disease parasite *Trypanosoma cruzi*. *Molecular Phylogenetics and Evolution* **41**, 209–221 (2006).
11. Kiyoshi, T. & Sota, T. Differentiation of the Dragonfly Genus Davidius (Odonata: Gomphidae) in Japan Inferred from Mitochondrial and Nuclear Gene Genealogies. *jzoo* **23**, 1–8 (2006).

12. Sota, T. & Hayashi, M. Comparative historical biogeography of Plateumaris leaf beetles (Coleoptera: Chrysomelidae) in Japan: interplay between fossil and molecular data. *Journal of Biogeography* **34**, 977–993 (2007).

13. Caccone, A. & Sbordon, V. Molecular Biogeography of Cave Life: A Study Using Mitochondrial Dna from Bathysciine Beetles. *Evolution* **55**, 122–130 (2001).

14. Percy, D. M., Page, R. D. M. & Cronk, Q. C. B. Plant–Insect Interactions: Double-Dating Associated Insect and Plant Lineages Reveals Asynchronous Radiations. *Systematic Biology* **53**, 120–127 (2004).

15. Pons, J. & Vogler, A. P. Complex Pattern of Coalescence and Fast Evolution of a Mitochondrial rRNA Pseudogene in a Recent Radiation of Tiger Beetles. *Molecular Biology and Evolution* **22**, 991–1000 (2005).

16. Wahlberg, N. That Awkward Age for Butterflies: Insights from the Age of the Butterfly Subfamily Nymphalinae (Lepidoptera: Nymphalidae). *Systematic Biology* **55**, 703–714 (2006).

17. Ruiz, C., Jordal, B. & Serrano, J. Molecular phylogeny of the tribe Sphodrini (Coleoptera: Carabidae) based on mitochondrial and nuclear markers. *Molecular Phylogenetics and Evolution* **50**, 44–58 (2009).

18. Gratton, P., Konopiński, M. K. & Sbordoni, V. Pleistocene evolutionary history of the Clouded Apollo (Parnassius mnemosyne): genetic signatures of climate cycles and a 'time-dependent' mitochondrial substitution rate. *Molecular Ecology* **17**, 4248–4262 (2008).

19. Patané, J. S. L., Weckstein, J. D., Aleixo, A. & Bates, J. M. Evolutionary history of *Ramphastos* toucans: Molecular phylogenetics, temporal diversification, and biogeography. *Molecular Phylogenetics and Evolution* **53**, 923–934 (2009).

20. Fourdrilis, S. *et al.* Mitochondrial DNA hyperdiversity and its potential causes in the marine periwinkle Melarhaphe neritoides (Mollusca: Gastropoda). *PeerJ* **4**, (2016).

21. Andújar, C., Serrano, J. & Gómez-Zurita, J. Winding up the molecular clock in the genus Carabus(Coleoptera: Carabidae): assessment of methodological decisions on rate and node age estimation. *BMC Evolutionary Biology* **12**, 40 (2012).

22. Marshall, D. C. *et al.* Inflation of Molecular Clock Rates and Dates: Molecular Phylogenetics, Biogeography, and Diversification of a Global Cicada Radiation from Australasia (Hemiptera: Cicadidae: Cicadettini). *Systematic Biology* **65**, 16–34 (2016).

23. García-Ríos, C. I., Pérez-Pérez, N. M., Fernández-López, J. & Fuentes, F. A. Calibrating the chitons (Mollusca: Polyplacophora) molecular clock with the mitochondrial DNA cytochrome C oxidase I gene. *Rev. biol. mar. oceanogr.* **49**, 193–207 (2014).

24. Marko, P. B. Fossil Calibration of Molecular Clocks and the Divergence Times of Geminate Species Pairs Separated by the Isthmus of Panama. *Molecular Biology and Evolution* **19**, 2005–2021 (2002).

25. Lavinia, P. D., Kerr, K. C. R., Tubaro, P. L., Hebert, P. D. N. & Lijtmaer, D. A. Calibrating the molecular clock beyond cytochrome b: assessing the evolutionary rate of COI in birds. *Journal of Avian Biology* **47**, 84–91 (2016).

26. Saltzwedel, H. von, Scheu, S. & Schaefer, I. Genetic structure and distribution of Parisotoma notabilis (Collembola) in Europe: Cryptic diversity, split of lineages and colonization patterns. *PLOS ONE* **12**, e0170909 (2017).

27. Hupało, K., Karaouzas, I., Mamos, T. & Grabowski, M. Molecular data suggest multiple origins and diversification times of freshwater gammarids on the Aegean archipelago. *Sci Rep* **10**, 19813 (2020).

28. Wang, H., Fan, X., Owada, M., Wang, M. & Nylin, S. Phylogeny, Systematics and Biogeography of the Genus Panolis (Lepidoptera: Noctuidae) Based on Morphological and Molecular Evidence. *PLOS ONE* **9**, e90598 (2014).

29. Khan, F. A. A., Phillips, C. D. & Baker, R. J. Timeframes of Speciation, Reticulation, and Hybridization in the Bulldog Bat Explained Through Phylogenetic Analyses of All Genetic Transmission Elements. *Systematic Biology* **63**, 96–110 (2014).

30. Foltz, D. W., Nguyen, A. T., Kiger, J. R. & Mah, C. L. Pleistocene speciation of sister taxa in a North Pacific clade of brooding sea stars (Leptasterias). *Mar Biol* **154**, 593–602 (2008).

31. Loeza-Quintana, T. & Adamowicz, S. J. Iterative Calibration: A Novel Approach for Calibrating the Molecular Clock Using Complex Geological Events. *J Mol Evol* **86**, 118–137 (2018).

32. Pellegrino, I. *et al.* Phylogeography and Pleistocene refugia of the Little Owl *A thene noctua* inferred from mt DNA sequence data. *Ibis* **156**, 639–657 (2014).

33. Ketmaier, V., Giusti, F. & Caccone, A. Molecular phylogeny and historical biogeography of the land snail genus *Solatopupa* (Pulmonata) in the peri-Tyrrhenian area. *Molecular Phylogenetics and Evolution* **39**, 439–451 (2006).

34. Schubart, C. D., Diesel, R. & Hedges, S. B. Rapid evolution to terrestrial life in Jamaican crabs. *Nature* **393**, 363–365 (1998).

35. Soares, P. *et al.* Correcting for Purifying Selection: An Improved Human Mitochondrial Molecular Clock. *Am J Hum Genet* **84**, 740–759 (2009).

36. Chevaldonné, P., Jollivet, D., Desbruyères, D., Lutz, R. & Vrijenhoek, R. Sister-species of eastern Pacific hydrothermal-vent worms (Ampharetidae, Alvinelidae, Vestimentifera) provide new mitochondrial clock calibration. *Cahiers de Biologie Marine* **43**, 367–370 (2002).
