# Supplementary material for: Quaternary Ice Ages Shaped Protists Phylogeography: The Case of Arcellinida in the Iberian Peninsula
Source: Mol Ecol. 2026 Jul 18;35(14):e70475. doi: 10.1111/mec.70475 (PMC13380319; doi:10.1111/mec.70475)
Supplement: Supplementary file 6 — Figure S1: Informative terrestrial OTUs in the Iberian Peninsula. (A) Percentage of molecular variance explained by ecoregion (AMOVA): Analysis of molecular variance (AMOVA) for each informative OTU, showing the percentage of molecular variance attributable to ecoregion. (B) PHd of each OTU per ecoregion: Results of the PHd analysis for the informative OTUs, summarized by ecoregion. Figure S2: Geographic distribution of each OTU present in the Mediterranean ecoregion of the Iberian Peninsula. Each panel shows a map with the localities where the corresponding OTU (number shown above) was detected. Points represent sampling localities and are coloured according to nucleotide diversity (π) estimated for that OTU in that locality, following the colour scale shown Figure S3: Geographic distribution of each OTU present in the Temperate ecoregion of the Iberian Peninsula. Each panel shows a map with the localities where the corresponding OTU (number shown above) was detected. Points represent sampling localities and are coloured according to nucleotide diversity (π) estimated for that OTU in that locality, following the colour scale shown Figure S4: Results of one‐way ANOVAs for environmental variables from WorldClim that differed significantly between the Mediterranean and Temperate ecoregions. Variables are ranked by F‐statistic and displayed as a horizontal bar plot. Variable names follow the WorldClim bioclimatic codes (bio). Figure S5: Relationship between haplotype richness and geographic range size for informative OTUs in the Mediterranean and Temperate ecoregions. Geographic range was estimated as the area (km2) of a convex hull enclosing all occurrence records for each OTU, based on coordinate data. Haplotype richness and range size were analysed separately for each ecoregion using Spearman's rank correlation. Points represent OTUs (Mediterranean: yellow triangles; Temperate: blue circles), with dashed lines showing fitted trends. Spearman's ρ and p‐values are ind [file MEC-35-e70475-s001.pdf]

Supplemental figures for:

Quaternary ice ages shaped protists phylogeography: the case of Arcellinida in the Iberian Peninsula

Rubén González-Miguéns, Emilio Cano, Enrique Lara

Figure	page
Fig. S1	2
Fig. S2	3
Fig. S3	4
Fig. S4	5
Fig. S5	6

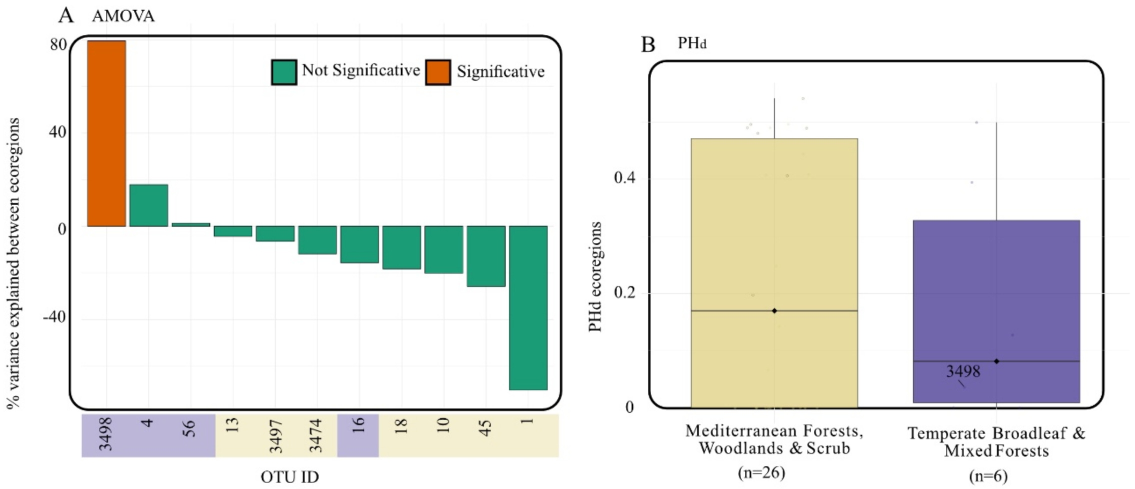

Figure S1. Informative terrestrial OTUs in the Iberian Peninsula. (A) Percentage of molecular variance explained by ecoregion (AMOVA): Analysis of molecular variance (AMOVA) for each informative OTU, showing the percentage of molecular variance attributable to ecoregion. (B) PH_d of each OTU per ecoregion: Results of the PH_d analysis for the informative OTUs, summarised by ecoregion.

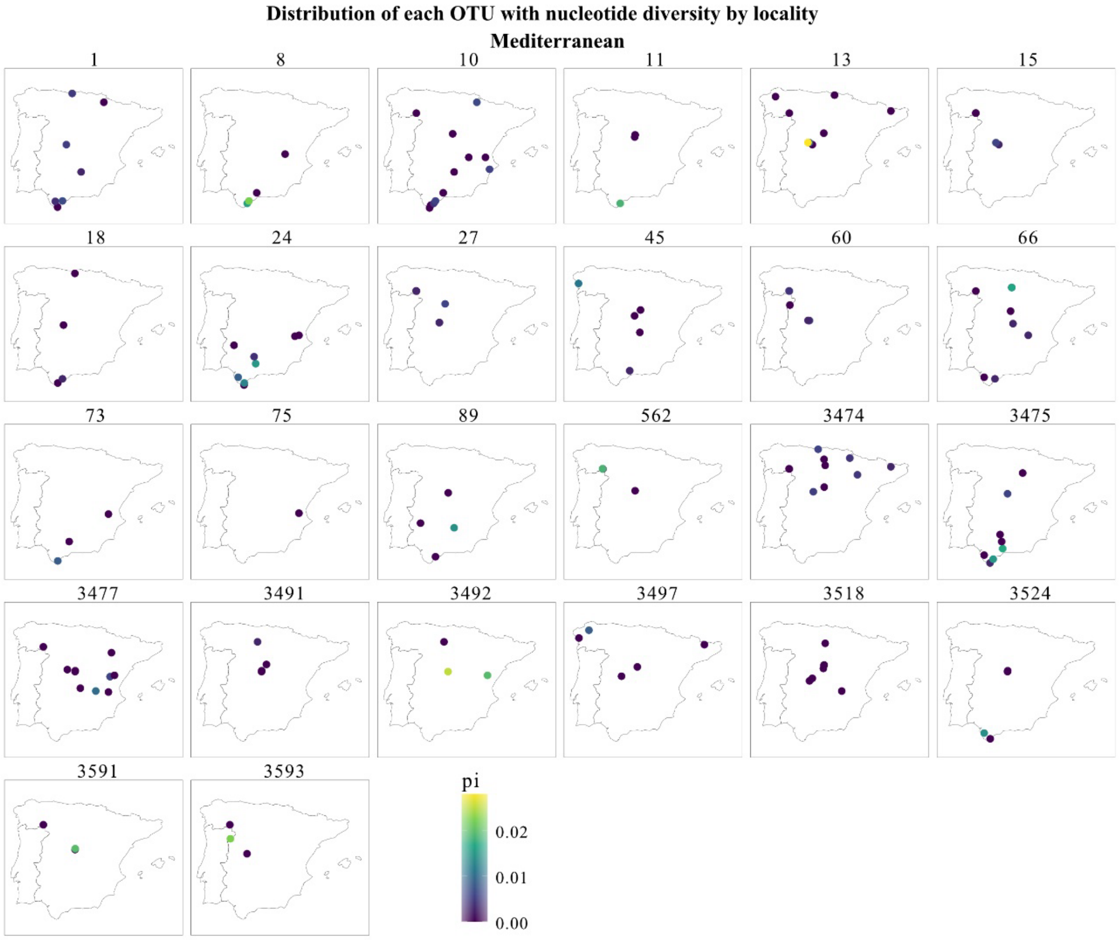

Figure S2. Geographic distribution of each OTU present in the Mediterranean ecoregion of the Iberian Peninsula. Each panel shows a map with the localities where the corresponding OTU (number shown above) was detected. Points represent sampling localities and are coloured according to nucleotide diversity (π) estimated for that OTU in that locality, following the colour scale shown

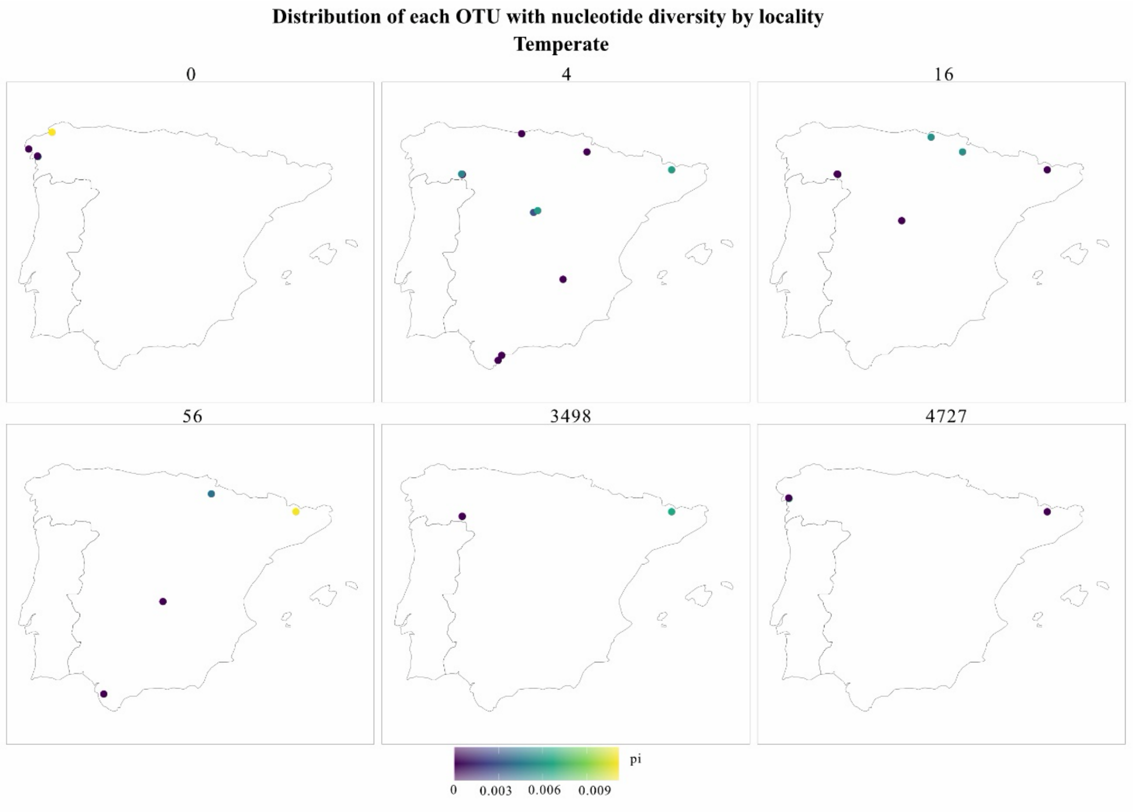

Figure S3. Geographic distribution of each OTU present in the Temperate ecoregion of the Iberian Peninsula. Each panel shows a map with the localities where the corresponding OTU (number shown above) was detected. Points represent sampling localities and are coloured according to nucleotide diversity (π) estimated for that OTU in that locality, following the colour scale shown

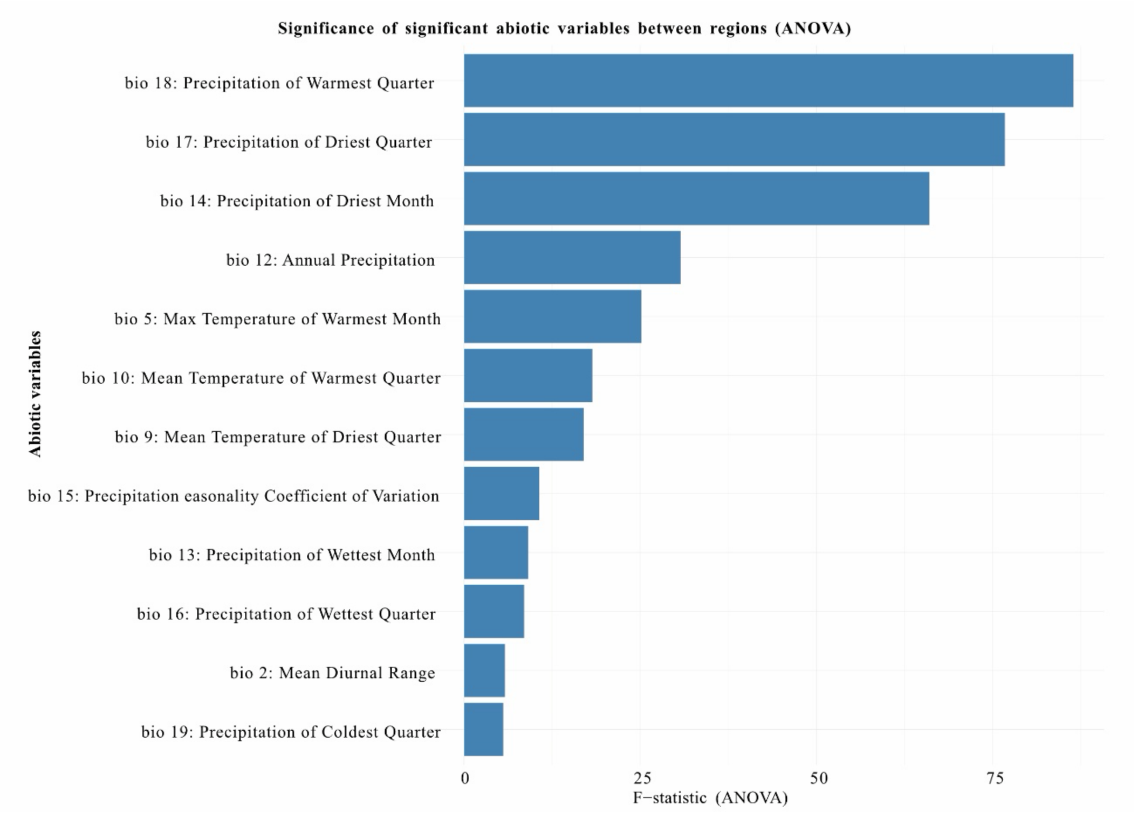

Figure S4. Results of one-way ANOVAs for environmental variables from WorldClim that differed significantly between the Mediterranean and Temperate ecoregions. Variables are ranked by F-statistic and displayed as a horizontal bar plot. Variable names follow the WorldClim bioclimatic codes (bio).

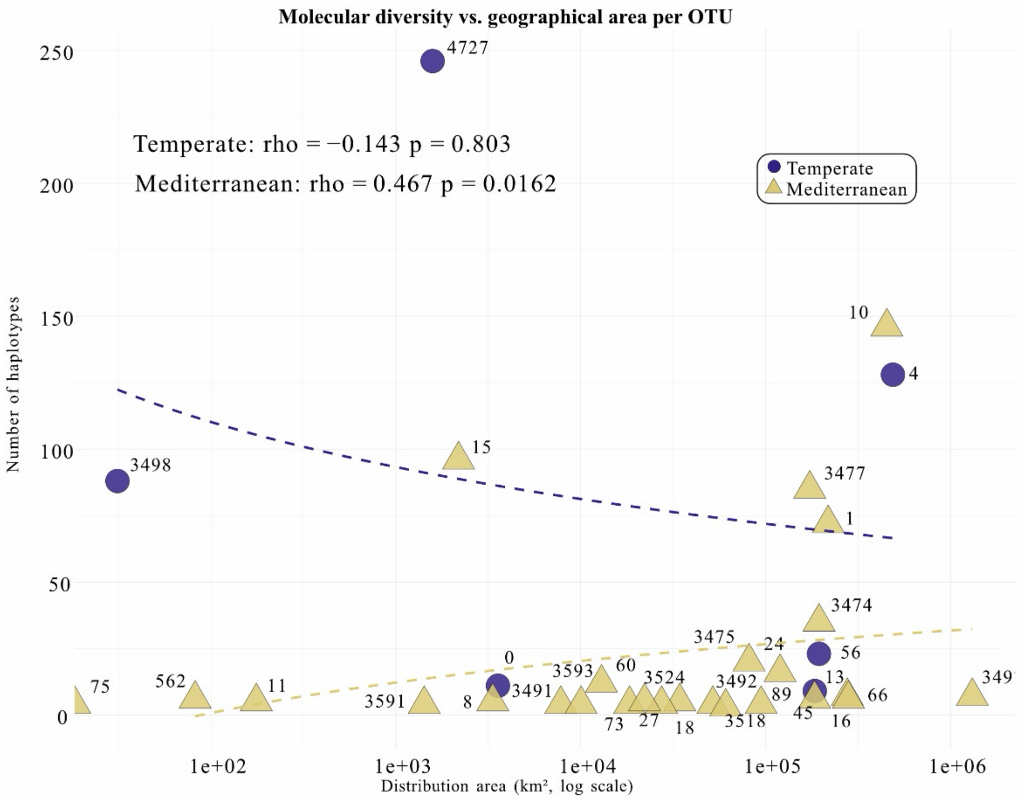

Figure S5. Relationship between haplotype richness and geographic range size for informative OTUs in the Mediterranean and Temperate ecoregions. Geographic range was estimated as the area (km²) of a convex hull enclosing all occurrence records for each OTU, based on coordinate data. Haplotype richness and range size were analysed separately for each ecoregion using Spearman's rank correlation. Points represent OTUs (Mediterranean: yellow triangles; Temperate: blue circles), with dashed lines showing fitted trends. Spearman's ρ and p-values are indicated for each ecoregion.
